# Renal angiomyolipoma-investigating radiological signs indicative of risk for bleeding

**DOI:** 10.1186/s13244-025-01957-z

**Published:** 2025-04-05

**Authors:** Jesper Swärd, Karl Bohlin, Olof Henrikson, Sven Lundstam, Ralph Peeker, Anna Grenabo Bergdahl

**Affiliations:** 1 Institute of Clinical Sciences, Sahlgrenska Academy, University of Gothenburg, Gothenburg, Sweden; 2 Department of Urology, Region Västra Götaland, NU-Hospital Group, Uddevalla, Sweden; 3 Department of Radiology, Region Västra Götaland, Sahlgrenska University Hospital, Gothenburg, Sweden; 4 Department of Urology, Region Västra Götaland, Sahlgrenska University Hospital, Gothenburg, Sweden; 5 Department of Oncology, Region Västra Götaland, Sahlgrenska University Hospital, Gothenburg, Sweden

**Keywords:** Kidney, Angiomyolipoma, Haemorrhage, Tomography (Spiral computed), Radiology (Interventional)

## Abstract

**Objectives:**

To compare imaging differences between bleeding and non-bleeding angiomyolipoma with respect to the proportion and attenuation of the angiomyogenic component and the occurrence and size of aneurysms.

**Materials and methods:**

CT scans and angiographies preceding 58 consecutive embolisations at two institutions from 1999 to 2018 were analysed retrospectively. Tumour volume was measured by contouring the angiomyolipoma on CT scans. The partial volume of the angiomyogenic component (blood vessels and smooth muscle relative to fatty tissue) was derived using attenuation threshold values measured in Hounsfield Units.

**Results:**

Bleeding angiomyolipoma exhibited a significantly higher proportion of angiomyogenic component (23%) than non-bleeding angiomyolipoma (8%) (*p* = 0.042). Angiomyolipoma with 0–5% angiomyogenic component had a lower risk of bleeding compared to those with ≥ 5% angiomyogenic component (13% vs 42%). Mean attenuation values of angiomyogenic components did not differ between bleeders and non-bleeders. Aneurysms were observed in 24% of angiomyolipoma during angiography. No statistically significant association was found between the occurrence of aneurysms and bleeding, neither when all aneurysms were included nor when only aneurysms ≥ 5 mm were considered. Tuberous sclerosis patients had larger tumours (11.4 cm vs 6.0 cm), but no significant difference in bleeding was observed (*p* = 0.53).

**Conclusions:**

A higher proportion of the angiomyogenic component in bleeding renal angiomyolipoma suggests a possible association with bleeding. Angiomyolipoma with less than 5% angiomyogenic components may represent a subgroup with a reduced risk of bleeding. Our findings do not confirm the widely accepted assumption that aneurysms significantly increase the risk of bleeding.

**Critical relevance statement:**

Measuring the angiomyogenic component in renal angiomyolipoma could help address current knowledge gaps and aid in the more efficient selection of patients for therapeutic interventions.

**Key Points:**

- Identifying risk factors for bleeding beyond tumour size is important.
- Very low angiomyogenic component tumours may have reduced bleeding risk.
- The presence of aneurysms may not significantly increase bleeding risk.
- Reporting angiomyogenic proportion on CT may aid in treatment decisions.

**Graphical Abstract:**

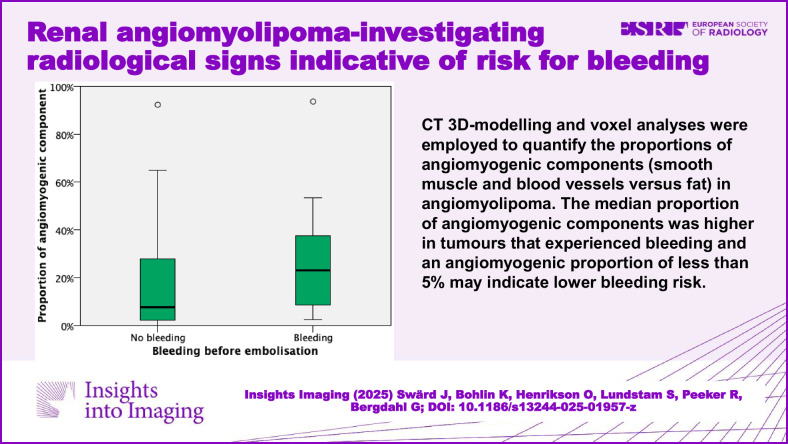

## Introduction

Spontaneous renal bleeding into the perinephric space is a rare but potentially life-threatening condition. Renal neoplasms are the most common aetiology, with angiomyolipoma predominating [1]. While most angiomyolipoma exhibit an indolent behaviour, our current understanding lacks precise markers to identify those at risk of spontaneous bleeding. The traditional four-centimetre size cut-off for prophylactic intervention is increasingly under scrutiny, and other indicators of bleeding risk are needed [2–4].

Angiomyolipoma are composed of blood vessels (angio), smooth muscle (myo) and fat (lipoma) in varying proportions. Computed tomography (CT) allows for the differentiation and quantitative assessment of fatty vs angiomyogenic components through three-dimensional (3D) modelling. Despite this, few studies have delved into tumour composition as a risk factor for bleeding, and no study has previously investigated whether differences in contrast attenuation of the angiomyogenic component influence bleeding risk.

Vessels in angiomyolipoma are eccentric and thick-walled [5]. Angiographies often reveal irregular, tortuous vessels with microaneurysms [6–10]. Notably, both the presence and size of aneurysms have been proposed as potential predictors of rupture [7–9]. These findings have since been widely referred to. However, existing studies on this subject typically only include a small number of bleeding cases.

Renal angiomyolipoma come in two types: the sporadic form and those linked to tuberous sclerosis complex (TSC). Sporadic ones are usually single, symptom-free, and more common in females. Conversely, TSC-associated ones often appear as multiple, bilateral tumours without a gender difference [11, 12]. However, scientific evidence regarding their correlation with bleeding remains limited [13].

The aim of this study is to examine radiological characteristics, including tumour tissue composition, contrast attenuation, and aneurysm presence, in patients with and without bleeding prior to embolisation.

## Materials and methods

**Fig. 1:**
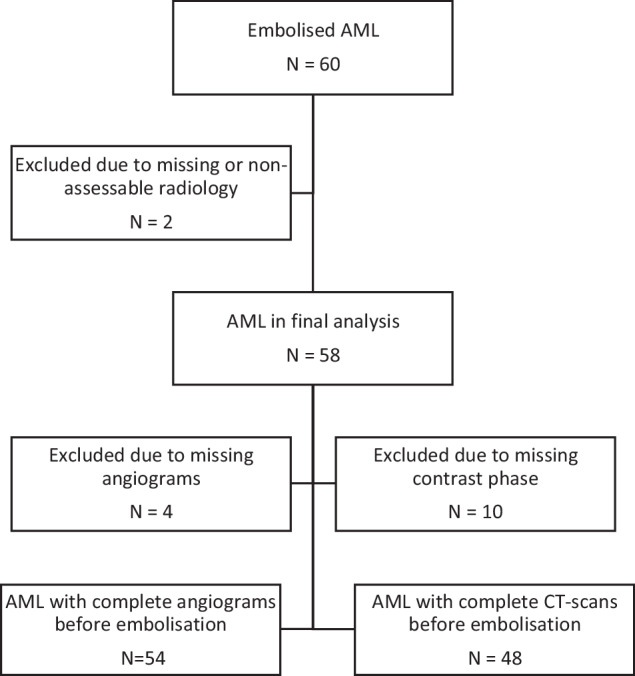
Flowchart of patients treated with selective arterial embolisation at Sahlgrenska University Hospital and NU Hospital Group between 1999 and 2018. Data per tumour. AML, Angiomyolipoma

This study retrospectively analysed 58 angiomyolipoma from 53 patients treated with first-time selective arterial embolisation between 1999 and 2018 at Sahlgrenska University Hospital and NU Hospital Group (Fig. 1). Patients were identified in the medical databases as previously described [14]. Two patients lacking pre-embolisation cross-sectional imaging were excluded. Ethical approval was granted (application 238-17; amendment 2020-01723) and the study adhered to the principles of the Declaration of Helsinki.

Angiomyolipoma were diagnosed via CT scans showing intra-tumoural fat (< −10 Hounsfield Units [HU]) without calcification or necrosis. Two radiologists (K.B. and O.H.) evaluated imaging data using dedicated software (AW-server 3.2 Ext 3.4, GE Healthcare).

### Tumour tissue composition

CT scans conducted prior to embolisation were used to evaluate tumour tissue composition. Technical details, including detector channels and slice thickness, are summarised in Table 1. Complete investigations (*n* = 48) were defined as those adequately visualising and contrast-enhancing the angiomyolipoma. The scans utilised various protocols, with assessments performed in the arterial (*n* = 2), corticomedullary (*n* = 17), and venous phases (*n* = 29).

**Table 1:** Characteristics of CT scanners and imaging reconstructions used in the assessment of the angiomyogenic component in 48 angiomyolipoma

		*n* = 48
Number of detector channels, *n* (%)	2–8	9 (19)
	16–40	10 (21)
	64–265	29 (60)
Thickness of CT-slices, mm, *n* (%)	0.62–1.5	5 (11)
	3	16 (33)
	5	26 (54)
	7.5	1 (2)

The investigations were performed between 1999 and 2018

Tumour volumes were estimated by manually outlining the tumour’s outer contour on axial slices using the “paint on slices” tool, followed by 3D reconstructions within a spherical region of interest (ROI). Partial volumes in specified HU intervals were determined using attenuation threshold values. Tissue exceeding 50 HU on contrast-enhanced scans was classified as angiomyogenic to exclude potential haematoma contributions.

The angiomyogenic component was calculated as the volume of contrast-enhancing tissue divided by the total tumour volume and reported in absolute (cm³) and proportional (%) terms.

### Tumour attenuation

To assess tumour enhancement, the mean HU value of the angiomyogenic component was calculated from contrast-enhanced scans for each tumour and compared with mean HU values from three kidney cortex ROIs, as well as the aorta and vena cava. To account for inter-patient variations in contrast phases, two ratios were derived: the mean HU of the angiomyogenic component relative to the kidney cortex and relative to the aorta and vena cava.

### Aneurysms

Angiograms were reviewed for aneurysms, defined as saccular or fusiform dilations. Aneurysm size was estimated using catheter references when angiograms lacked calibration. CT-derived aneurysm measurements were conducted on multiplanar reconstructions.

### TSC-status

Information on TSC status was extracted from medical records.

### Statistics

Data were analysed using SPSS version 29. Mann–Whitney *U*-tests were used for continuous variables, while Chi-square or Fisher’s exact tests compared categorical data. A *p*-value of < 0.05 was considered statistically significant.

## Results

In this study, 53 patients with a total of 58 embolised angiomyolipoma were included. Indications for embolisation were either bleeding or growth during active surveillance. Among the 58 angiomyolipoma, 24 (41%) had experienced bleeding prior to embolisation (Table 2). Complete investigations to assess the angiomyogenic component were available for 48 angiomyolipoma (16 with bleeding and 32 without bleeding) (Table 3). Angiograms for evaluating aneurysms were available for 54 angiomyolipoma (21 with bleeding and 33 without bleeding) (Table 4).

**Table 2:** Baseline characteristics of angiomyolipoma with and without a history of bleeding, treated with selective arterial embolisation at Sahlgrenska University Hospital and NU Hospital Group between 1999 and 2018 (data per tumour)

		Bleeding *n* = 24	No bleeding *n* = 34
Age at diagnosis, years, median (IQR)		44 (33–59)	46 (34–59)
Gender, *n* (%)	Male	3 (43)	4 (57)
	Female	21 (41)	30 (59)
TSC, *n* (%),	Yes	6 (50)	6 (50)
	No	18 (39)	28 (61)
Multifocality, *n* (%)	Single	15 (44)	19 (56)
	Multiple	9 (38)	15 (63)
Diameter at diagnosis, median (IQR)		6.5 (5.0–9.9)	5.9 (5.0–8.1)

*TSC* tuberous sclerosis complex

**Table 3:** Characteristics of 48 angiomyolipomas with adequate radiological images to assess for angiomyogenic components, treated with selective arterial embolisation at Sahlgrenska University Hospital and NU Hospital Group between 1999 and 2018

		Bleeding *n* = 16	No bleeding *n* = 32
Age at SAE, years, median (IQR)		49 (37–65)	49 (41–63)
Gender, *n* (%)	Male	2 (33)	4 (67)
	Female	14 (33)	28 (67)
TSC, *n* (%)	Yes	2 (25)	6 (75)
	No	14 (35)	26 (65)
Diameter at diagnosis, median (IQR)		6.0 (5.0–8.1)	5.9 (5.0–8.1)
Diameter at SAE, cm, median (IQR)		6.5 (5.1–8.8)	7.5 (5.4–9.6)
Volume, cm^3^, median (IQR)		111 (69–253)	132 (65–255)
Volume of angiomyogenic component, median, cm^3^, (IQR)		27 (8–68)	8 (3–33)
Proportion angiomyogenic component median, (%) (IQR)		23 (9–38)	8 (2–28)
Bleeding events categorised by angiomyogenic component, *n* (%)			
	0–5%	2 (13)	13 (87)
	≥ 5%	14 (42)	19 (58)

Angiomyolipomas are categorised based on the presence or absence of bleeding events prior to embolisation. The data is presented on a per-tumour basis

*TSC* tuberous sclerosis complex, *SAE* selective arterial embolisation

**Table 4:** Clinical characteristics and CT-findings of 54 angiomyolipoma treated with selective arterial embolisation at Sahlgrenska University Hospital and NU Hospital Group between 1999 and 2018, in relation to aneurysm occurrence on angiograms (data provided on a per-tumour basis)

		Aneurysms *n* = 13	No aneurysms *n* = 41
Age at diagnosis, years (median IQR)		30 (20–45)	53 (42–64)
Gender, *n* (%)	Male	0 (0)	6 (100)
	Female	13 (27)	35 (73)
TSC, *n* (%)	Yes	7 (70)	3 (30)
	No	6 (14)	38 (86)
Size at diagnosis, cm, median (IQR)		6.7 (5.7–10.0)	6.0 (4.9–8.2)
Size at SAE, cm, median (IQR)		8.6 (6.0–12.4)	6.9 (5.1–9.4)
Bleeding at diagnosis, *n* (%)	Yes	7 (33)	14 (67)
	No	6 (18)	27 (82)
Proportion angiomyogenic component at SAE, median, % (IQR)		29^a^ (20–52)	8^b^ (3–24)

*TSC* tuberous sclerosis complex, *SAE* selective arterial embolisation

^a^ Based on 10 patients with contrast-enhanced CT

^b^ Based on 38 patients with contrast-enhanced CT

### Tumour tissue composition (*n* = 48)

Bleeding angiomyolipoma were slightly smaller at embolisation in terms of axial diameters (6.5 cm vs 7.5 cm) and total volumes (111 cm^3^ vs 132 cm³), though these differences were not statistically significant (*p* = 0.69) (Table 3). Most angiomyolipoma contained more fatty tissue than blood vessels or smooth muscle.

**Fig. 2:**
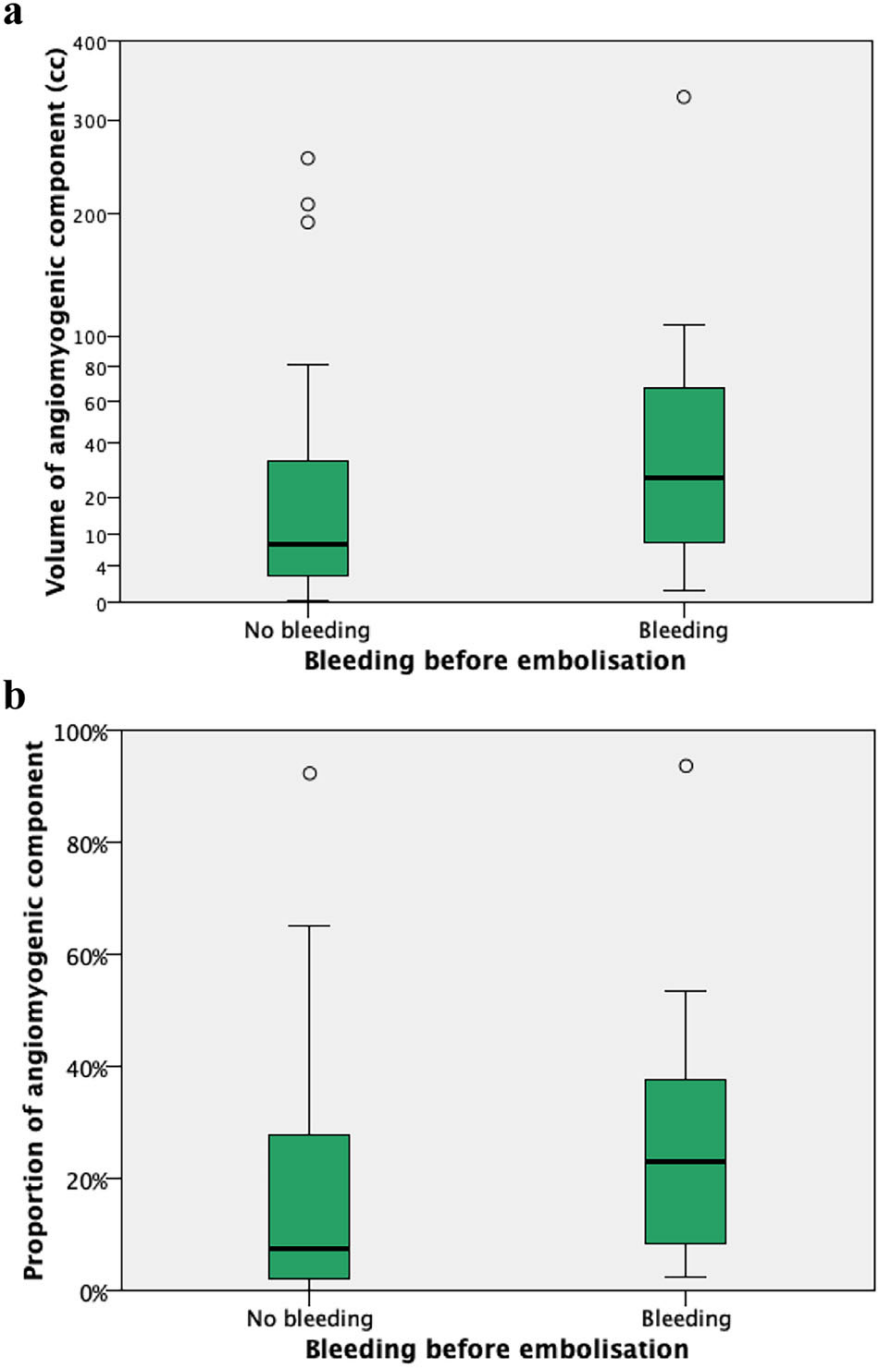
**a** Distribution of the volume of the angiomyogenic component, encompassing blood vessels and muscle, in 48 angiomyolipoma, with or without bleeding, before embolisation. Note the non-linear scale on the *y*-axis, which better highlights differences in the box plots compared to a linear scale. **b** Distribution of the proportion of the angiomyogenic component in the same tumours

**Fig. 3:**
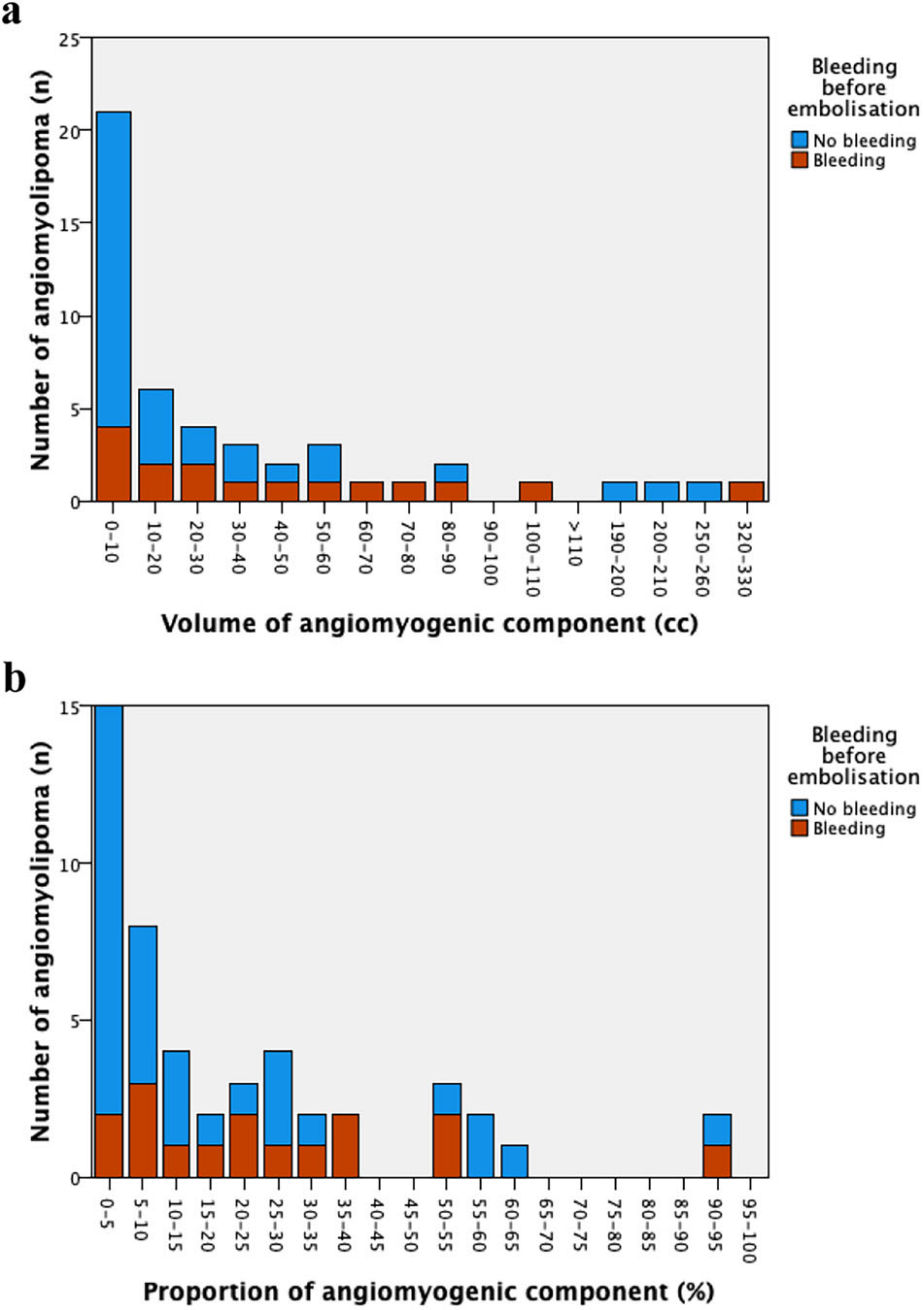
**a** Number of angiomyolipoma, with or without bleeding before embolisation, grouped by *volume* of the angiomyogenic component (in intervals of 10 cm^3^). Note that the intervals to the right of > 110 cm³ on the *x*-axis are not consistent in scale. **b** Number of angiomyolipoma, with or without bleeding before embolisation, grouped by *proportion* of the angiomyogenic component (in intervals of 5%)

The median angiomyogenic component volume was 12 cm³ (3–50), with bleeding tumours showing a higher median volume (27 cm³) compared to non-bleeding ones (8 cm³), although this difference was not significant (*p* = 0.11) (Fig. 2a). Non-bleeding tumours were predominantly in the 0–10 cm³ category of angiomyogenic component volume, where only 19% (4/21) bled (Fig. 3a).

The median angiomyogenic component proportion was 10% (3–31), with bleeding tumours showing a higher median proportion (23%) compared to non-bleeding ones (8%) (*p* = 0.042) (Fig. 2b). Most non-bleeding tumours fell within the 0–5% and 5–10% proportion categories, where only 13% (2/15) bled in the lowest group (Fig. 3b).

Comparing bleeding in the 0–5% and 5–100% proportion groups showed weak significance (13% vs 42%, *p* = 0.048). Exploratory comparisons (0–10% vs 10–100% and 0–15% vs 15–100%) revealed no significant differences.

### Tumour attenuation (*n* = 48)

The median attenuation of the angiomyogenic component was similar between bleeding (73 HU, IQR: 67–76) and non-bleeding angiomyolipoma (77 HU, IQR: 70–82). After adjusting for kidney cortex attenuation, the median ratios were nearly identical for non-bleeding (0.55, IQR: 0.47–0.63) and bleeding cases (0.56, IQR: 0.42–0.62) (*p* = 0.79). Adjustments using aorta and vena cava attenuation yielded similar results, with median ratios of 0.61 (0.54–0.70) for non-bleeding and 0.60 (0.48–0.67) for bleeding cases (*p* = 0.21). No significant differences in attenuation, as a measure of contrast enhancement, were observed between bleeders and non-bleeders.

### Aneurysms (*n* = 54)

Aneurysms were identified in 24% (13/54) of angiomyolipoma on angiograms (Table 4), all in female patients. The median aneurysm size was 8 mm (5–10), with multiple aneurysms observed in 10 cases, with a median of 3 per tumour (2–5). Aneurysms ≥ 5 mm were present in 11 tumours, six of which were associated with bleeding.

In 50% (5/10) of cases with pre-embolisation contrast-enhanced CT scans, aneurysms seen on angiograms were detected beforehand, though these CT protocols were not optimised for angiography.

Bleeding was observed in 54% (7/13) of angiomyolipoma with aneurysms, compared to 34% (14/41) without aneurysms (*p* = 0.33). Restricting the analysis to aneurysms ≥ 5 mm, bleeding rates were 55% (6/11) vs 35% (15/43), with no statistically significant difference (*p* = 0.31).

### Tumour multifocality (*n* = 58)

Of the 58 angiomyolipoma, 34 (59%) were single and 24 (41%) were multiple. Having multiple angiomyolipoma did not increase the risk of bleeding compared to single tumours (*p* = 0.61).

### TSC-status (*n* = 58)

Twelve of 58 angiomyolipoma (21%) were associated with TSC, with nearly all TSC cases being multiple (92%, 11/12) compared to 28% (13/46) in sporadic cases. TSC-associated angiomyolipoma were significantly larger than sporadic ones, with median diameters (10.0 cm vs 6.0 cm), volumes (288 cm³ vs 104 cm³), and angiomyogenic component volumes (150 cm³ vs 9 cm³); all *p* < 0.001. The median proportion of the angiomyogenic component was higher in TSC-associated cases (40% vs 9%), though not statistically significant (*p* = 0.11).

Aneurysms were more common in TSC-associated angiomyolipoma (70%, 7/10) compared to sporadic cases (18%, 6/34, *p* < 0.001). Median aneurysm size (10 mm vs 5.5 mm) and number (4 vs 2) were also greater in TSC cases. There was no significant difference in bleeding rates prior to embolisation between TSC-associated and sporadic angiomyolipoma (*p* = 0.53) (Table 2).

## Discussion

Identifying risk factors for predicting haemorrhage in patients with angiomyolipoma represents a challenge. To the best of our knowledge, this study includes the largest cohort of patients with comprehensive radiological mapping of angiomyolipoma, some of whom experienced a bleeding event prior to selective arterial embolisation. We demonstrate that bleeding angiomyolipoma exhibits a significantly higher median proportion of angiomyogenic components (23% vs 8%), comprising vessels and smooth muscle. However, contrary to previous beliefs, aneurysm occurrence does not seem to be strongly associated with an increased risk of bleeding.

Albeit being the most common aetiology of spontaneous renal haemorrhage into the retroperitoneal space, most angiomyolipoma are indolent. Two reviews exploring the current understanding of spontaneous renal haemorrhage documented 165 cases between 1985 and 1999 and 102 cases between 2000 and 2017 [1, 15]. Of these, 29% and 42%, respectively, were found to be caused by angiomyolipoma. Size has commonly been considered a major risk factor for bleeding, but its significance is increasingly questioned [2, 4, 16]. Studies investigating other risk factors, such as the proportions of fat and angiomyogenic components, are scant. In 2005, Rimon investigated a three-tiered grading system for vascularity, primarily based on angiographies [10]. The study suggested an elevated risk of bleeding in angiomyolipoma with heightened vascularity, although statistical significance was not achieved. Subsequent reports include Combs’s recent findings, demonstrating a > 50% angiomyogenic component in all five ruptured angiomyolipomas within their series [17]. Studies on the impact of absolute volumes of angiomyogenic components are hitherto lacking.

The probability of bleeding was low among angiomyolipoma with smaller proportions (< 5%) and absolute volumes (< 10 cm^3)^ of angiomyogenic components. This finding may, if reproduced, have clinical implications as a large proportion of angiomyolipoma probably exhibit this composition (fat-rich). In this selected cohort, one-third of all angiomyolipoma had a proportion of angiomyogenic components of less than 5%. It may be assumed that this share is even larger in an unselected population of patients with angiomyolipoma. It could then be of clinical interest to state the angiomyogenic proportion in the radiology report, as it may aid in treatment decisions. However, these concepts require further studies before any recommendations can be made.

Small renal masses are frequent incidental findings on imaging. Some of these represent asymptomatic angiomyolipoma that require active surveillance or prophylactic embolisation. Defining a low-risk group for bleeding events is thus clinically relevant in terms of avoiding overtreatment. However, differentiating tumours with 0–5% angiomyogenic component from those with ≥ 5% requires precise identification on CT scans. Manual tumour contouring is labour-intensive but crucial for detecting subtle differences in tumour content. Its clinical utility may therefore be limited, though AI-driven tools for contouring hold promise for future clinical applications.

We hypothesised that the degree of attenuation within the angiomyogenic component, as a measure of contrast enhancement, would be important for bleeding risk. However, no such association was confirmed. To our knowledge, this has not been studied previously.

Aneurysm formation, especially larger aneurysms, is proposed to be associated with an increased risk of bleeding. Yamakado established this idea based on eight bleeding angiomyolipoma, all of which had aneurysms ≥ 5 mm [7]. However, 14 of 21 non-bleeding angiomyolipoma revealed aneurysm formation, including four ≥ 5 mm in size [7]. Champagnac demonstrated aneurysms in four of five haemorrhagic angiomyolipoma [8]. The mean diameter of the largest aneurysm was 9.5 mm compared to 3.9 mm in 17 unruptured angiomyolipoma. McQueen reported aneurysms in four of five bleeding angiomyolipoma, in contrast to 6 of 22 tumours without bleeding [18]. They found no significant difference in mean aneurysm size between the groups. Bardin reported a significant difference in the presence of aneurysms in a series of six bleeding and 17 non-bleeding angiomyolipoma [9]. In contrast, Rimon identified merely five aneurysms among 13 cases of bleeding angiomyolipoma, with only three surpassing the 5 mm threshold [10]. Our finding of aneurysms in 24% is in the lower range compared with previous reports: 13%, 35%, 39%, 40%, and 76% of tumours [6–10]. We found no significant difference regarding aneurysm occurrence in 21 bleeding and 33 non-bleeding tumours. Furthermore, we found no significant difference in bleeding between aneurysms measuring < 5 and ≥ 5 mm.

Consequently, aneurysm formation may not be such a decisive factor for bleeding events as previously thought. Another concern is that reliable aneurysm mapping requires invasive angiographies, as illustrated by our finding that only 50% of aneurysms were identified on CT scans. However, this detection rate might have been higher if all scans had been performed using a specific angiographic protocol. Only one study has explored aneurysms diagnosed via CT angiography, without confirmation through traditional angiographies [19]. This study, involving six bleeding and 28 non-bleeding angiomyolipoma, found no significant correlation between aneurysm size measured on CT and rupture. Future studies should ideally focus on aneurysms in angiomyolipoma identified by CT angiography, rather than traditional invasive methods.

It is hypothesised that individuals with TSC are more prone to vascular irregularities due to uncontrolled activation of mTOR and its effects on angiogenesis [5]. Aneurysm formation differed and was more common, larger, and often multiple in TSC patients. Whether this higher rate is due to TSC itself or reflects the fact that TSC-associated tumours were larger and had significantly greater partial volumes of angiomyogenic components remains unknown. Only two studies have compared TSC-associated angiomyolipoma to sporadic ones regarding angiomyogenic content and aneurysms. Rimon classified most TSC-associated angiomyolipoma as having heightened vascular complexity (*n* = 7), while Champagnac observed decreased fat content (*n* = 36) without significant differences in aneurysm incidence [8, 10].

This study has several limitations. Due to its retrospective exploratory design, no predefined statistical analysis plan was established. While this may raise concerns about drawing conclusions based on the statistics performed, we believe that the present study contributes to existing knowledge by the novel in-depth assessment of angiomyolipoma imaging.

All patients included in this study were selected to undergo an interventional procedure. Hence, a patient selection has taken place. This must be taken into consideration before applying the presented concepts to an unselected group of angiomyolipoma patients.

Given the rarity of the disorder, angiomyolipoma cases were collected over a twenty-year period. The imaging investigations were performed on a range of scanners with varying technical specifications and were reconstructed with different slice thicknesses. Additionally, contrast phases varied across the scans; however, in our view, the angiomyogenic component could be adequately outlined in the selected 48 cases, despite these differences in contrast phase.

Lastly, an unspecified number of patients with sporadic angiomyolipoma likely have undiagnosed TSC, as is the case in all other studies within the field.

## Conclusions

The higher proportion of the angiomyogenic component in bleeding angiomyolipoma suggests a potential link to haemorrhage and could be an important factor to consider when deciding on intervention, particularly as the emphasis on absolute size is being re-evaluated. Angiomyolipoma with a very low angiomyogenic component may represent a clinically relevant subgroup with a lower risk of spontaneous bleeding.

Our findings do not confirm the widely accepted notion that aneurysms, especially those 5 mm or larger, significantly increase bleeding risk.

## Data Availability

Data is available on request from Jesper Swärd (jesper.sward@gu.se).

## Abbreviations

3D: Three-dimensional
CT: Computed tomography
ROI: Region of interest
TSC: Tuberous sclerosis complex
